# Predictors of job satisfaction among German teachers during the SARS-CoV-2 pandemic

**DOI:** 10.1093/eurpub/ckac131.272

**Published:** 2022-10-25

**Authors:** T Dicks, V Eggert, C Köstner, T Beutel, K Kalo, C Zähme, S Letzel, P Dietz

**Affiliations:** 1 Institute of Occupational, Social and Environmental Medicine, University Medical Center of the University of Mainz, Mainz, Germany; 2 Institute of Teachers’ Health, University Medical Center of the Johannes Gutenberg University of Mainz, Mainz, Germany; 3 Department of Sports Medicine, Disease Prevention and Rehabilitation, Johannes Gutenberg University Mainz, Mainz, Germany

## Abstract

**Background:**

To contribute the containment of the infections during the SARS-COV-2 pandemic, changes in working conditions occurred worldwide. In the school context, teaching was changed several times to distance learning and teachers were forced to work from home. This increasing spatial separation between work and private life increased the potential for conflicts. Based on the theoretical assumption that stressors worsen job satisfaction and resources increase satisfaction, the aim of our study was to identify the predictors of teachers’ job satisfaction during the pandemic. The focus on job satisfaction is interesting because the concept is often related to health-related aspects from a public health perspective.

**Methods:**

A nationwide cross-sectional survey was conducted among German teachers in March 2021. After data cleaning, 31,089 participants were included in the analyses. The survey consisted of established instruments (e.g., COPSOQ) and self-developed items if necessary. A multiple linear regression was performed to predict teachers’ job satisfaction by stepwise inclusion of sociodemographic, work-related and covid-specific variables.

**Results:**

Overall, the regression revealed that especially work-related variables were strong predictors of job satisfaction. The analyses showed that higher levels of meaning of work, autonomy and predictability of work increased job satisfaction. In contrast, increased emotional stress, feelings of unfair treatment, and work-privacy conflicts deteriorated job satisfaction.

**Conclusions:**

The present study identified important predictors of job satisfaction which may be used to derive specific recommendations for improving teachers’ job satisfaction during the SARS-CoV-2 pandemic. The relevance of job satisfaction for the scientific and public discourse becomes apparent because it was closely related to teachers’ somatic and mental health.

**Key messages:**

• Teachers’ job satisfaction during the pandemic can be further improved by making appropriate adjustments, particularly in work-specific requirements.

• Improving job satisfaction significantly contributes to promoting teachers’ health.

